# Multi-Omics Analysis Reveals That Alkaline Mineral Complex Reshapes Rumen Mucosal Microbiota and Metabolites and Enhances Rumen Epithelial Barrier Function in Fattening Cattle

**DOI:** 10.3390/ani16060992

**Published:** 2026-03-22

**Authors:** Xingyu Liu, Li Gu, Jia Li, Xiaowan Liu, Quan Mo, Liping Gou, Yixin Wang, Jiancheng Qi, Zhicai Zuo

**Affiliations:** Key Laboratory of Animal Disease and Human Health of Sichuan Province, College of Veterinary Medicine, Sichuan Agricultural University, 211 Huimin Road, Wenjiang District, Chengdu 611130, China; liuxingyu202510@126.com (X.L.); ligu19992025@163.com (L.G.); pengu1nnn@126.com (J.L.); guoguowan1990@163.com (X.L.); 15130@sicau.edu.cn (Q.M.); glping0827@163.com (L.G.); yixinwang21@163.com (Y.W.)

**Keywords:** cattle, AMC, rumen, epithelial barrier, tight junction protein, multi-omics

## Abstract

High-concentrate diets are widely used to enhance growth performance in fattening beef cattle; however, they often compromise rumen epithelial integrity, increasing the risk of rumenitis and systemic inflammation. Supplementation with alkaline mineral complex (AMC) has been shown to alleviate these adverse effects, although the underlying mechanisms remain largely unexplored. In this study, AMC supplementation was associated with improved rumen epithelial integrity and remodeling of the rumen microbiota, characterized by a reduction in *Bacteroidota* and *Prevotella*, and an enrichment of *Sarcina* sp. *DSM11001* and *Fibrobacter* spp., with the latter identified as a key microbial biomarker in the AMC group. Integrated metabolomic and transcriptomic analyses revealed activation of the tryptophan metabolism pathway and accumulation of several anti-inflammatory metabolites, including sulfinpyrazone, Thr-Leu, and 4-guanidinobutyric acid. These metabolomic changes were correlated with the upregulation of tight junction pathways and increased expression of related proteins, which in turn were associated with enhanced epithelial barrier integrity and reduced systemic inflammation in the AMC group. Collectively, these findings suggest that AMC supplementation may protect rumen epithelial integrity by modulating the microbial community and altering ruminal metabolite profiles. This study provides insights into nutritional strategies to prevent epithelial damage under high-concentrate feeding conditions and support the potential use of AMC to maintain rumen health in fattening cattle.

## Simple Summary

Feeding beef cattle high-concentrate diets boosts growth but can harm their rumen health, leading to inflammation. This study tested whether adding an alkaline mineral complex (AMC) to their feed could protect them. We found that AMC worked by positively changing the balance of microbes in the rumen. These changes increased the production of natural anti-inflammatory substances and strengthened the rumen lining. As a result, cattle fed AMC showed fewer signs of body-wide inflammation and better overall health. Our research provides a scientific basis for using this nutritional supplement to keep cattle healthier during intensive fattening, which is important for both animal welfare and sustainable farming.

## 1. Introduction

The rumen is a vital digestive organ in ruminants and serves as the primary site for microbial fermentation that produces volatile fatty acids [1]. Its diverse and symbiotic microbial community supports a wide array of metabolic functions, directly influencing both animal performance and host health [2]. To enhance fattening efficiency, meat-producing ruminants are often fed high-energy grain diets. While such diets improve production performance, they also pose a threat to rumen health by inducing microbial dysbiosis [3,4]. This dysbiosis can disrupt ruminal fermentation and metabolic processes, compromise the integrity of the rumen epithelial barrier, and ultimately impair animal health and reduce farm profitability [5,6].

To maintain rumen health and epithelial barrier function, dietary additives are commonly incorporated into ruminant diets. For example, sodium bicarbonate helps neutralize fermentation acids and reduce epithelial damage [7], while monensin modulates microbial composition and fermentation patterns, thereby supporting epithelial repair under high-concentrate feeding conditions [8,9]. Although yeast can effectively restore gastrointestinal microbial balance, especially when animals face digestive disorders or stress [10,11], its effects on growth performance in ruminants remain inconsistent due to strain specificity, limiting its reliable application in production [12]. Moreover, conventional additives present notable limitations. Sodium bicarbonate provides only temporary acid neutralization and, when included at levels exceeding 12.5 g/kg dry matter (DM), fails to offer sustained buffering while potentially reducing feed intake and rumination [13,14]. Monensin, despite its efficacy, has raised concerns regarding antibiotic resistance, prompting regulatory restrictions in both the European Union [15] and the United States [16] on its use in food-producing animals. These limitations underscore the urgent need for novel feed additives that can effectively alleviate ruminal epithelial injury and preserve barrier function without compromising the growth performance of beef cattle.

The alkaline mineral complex (AMC) contains several probiotics, including *Bacillus subtilis* (5 × 10^7^ CFU/g) and *Bacillus licheniformis* (1 × 10^8^ CFU/g), along with mineral components such as sodium, potassium, zinc, germanium, and metasilicate, as detailed in Supplementary Table S13 [17,18]. These probiotics help regulate gastrointestinal microbiota, suppress pathogenic bacteria, and enhance host immunity and disease resistance [19,20]. The mineral constituents contribute to maintaining extracellular fluid osmolality and acid-base balance, activating enzymatic activity, supporting immune function [21], and exerting anti-inflammatory and metabolism-promoting effects [22,23]. Additionally, AMC possesses alkaline buffering capacity and has been shown to alleviate gastric mucosal damage induced by acidic substances such as indomethacin [24]. It has also been safely and stably applied as a non-specific immune activator to promote growth performance in pigs [25]. Collectively, these attributes suggest that AMC may offer effective acid-buffering and neutralizing capabilities and thus hold potential for alleviating rumen epithelial injury and supporting rumen health in fattening beef cattle.

Therefore, this study investigated the effects of dietary supplementation with AMC during the fattening phase on rumen epithelial damage and rumen health in beef cattle. Clinical symptoms in fattening cattle were systematically monitored, and histopathological examinations, combined with multi-omics analyses, were employed to assess the effects of AMC on rumen epithelial microbiota composition, metabolic pathways, and barrier function, while further exploring its potential underlying molecular mechanisms. This research provides a novel strategy for maintaining rumen health in beef cattle and supports the sustainable development of the beef cattle industry.

## 2. Materials and Methods

### 2.1. Animal Treatments and Experimental Diets

**Figure 1:**
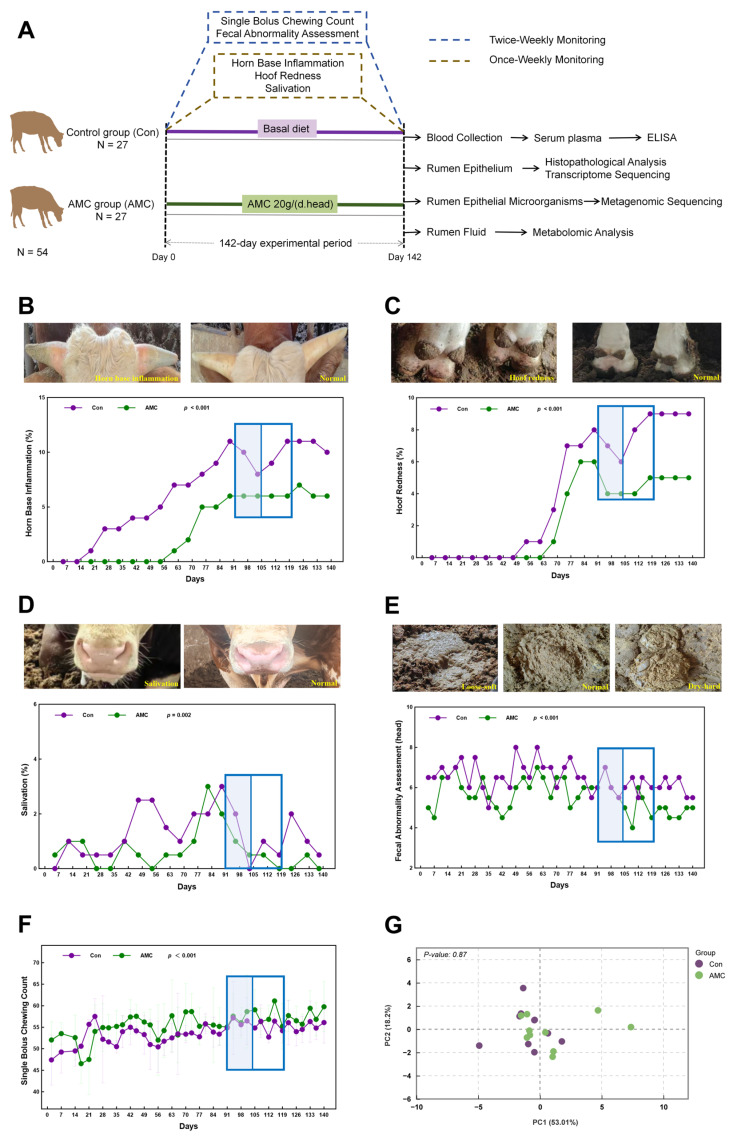
Experimental design and clinical performance of fattening beef cattle. (**A**): Schematic diagram illustrating the experimental design and procedures of this study; (**B**–**F**): Dot-line plots showing the clinical performance of fattening beef cattle throughout the 142-day trial, including horn-base inflammation (**B**), hoof redness (**C**), excessive salivation (**D**), abnormal feces (**E**), and single-bolus chewing counts (**F**); (**G**): Principal component analysis score plot showing the white blood cell count; In (**B**–**G**), data are presented as mean ± standard deviation and were analyzed using GLMM (**B**–**F**). Blue box: A trend of first decreasing and then increasing (from day 91 to day 119 of the experiment). Shaded area within the blue box: Diet adjustment period (from day 91 to day 104 of the experiment).

All animal procedures were conducted under the supervision of veterinarians at Caijiashan Livestock Breeding Co., Ltd. (Dazhou, China), a large-scale beef cattle operation located in Quxian County, Dazhou City, Sichuan Province, China. A total of 54 healthy Simmental crossbred bulls in the finishing phase, with comparable body condition, were randomly assigned to two groups (Con and AMC, N = 27 per group) using a completely randomized design. Randomization was performed using a random-number table generated by an independent researcher who was not involved in the animal management or data collection. One animal in the Con group died on day 54 due to extreme summer heat, resulting in final group sizes of 26 for Con and 27 for AMC by the end of the 142 day trial. Cattle in both groups were housed in well-ventilated pens with ad libitum access to water and fed a total mixed ration (TMR) formulated to meet the nutritional requirements of finishing beef cattle according to the NRC. The diet composition is detailed in Supplementary Table S1, with a concentrate-to-forage ratio of approximately 6:4. The AMC product was provided by Beijing Jnnail Biotechnology Co., Ltd. (Product Code: Q/NEL002-2019). Throughout the 142-day trial, the Con group received 0 g/day of AMC, while the AMC group received 20 g/day. The dosage was determined based on the feeding regimen recommended by the manufacturer. The AMC was precisely weighed, premixed with the concentrate, and then uniformly blended into the TMR. All cattle were fed twice daily at 9:30 AM and 4:30 PM. Due to market-related factors, the scheduled slaughter date was delayed, necessitating a mid-experiment adjustment to the feed formulation. The basal diet composition for days 91 to 104 is provided in Supplementary Table S12. The experimental design and timeline are summarized in Figure 1.

### 2.2. Quantification of Single-Bolus Chewing Behavior

Rumination behavior is a critical indicator of digestive health in cattle [26]. In this study, single-bolus chewing during each rumination event was quantified using a standardized visual counting method. For each observation, 10 animals were randomly selected from each group. Two trained observers, who were blinded to treatment allocation, simultaneously recorded the number of chews per bolus for each animal. Throughout the observation period, the observers remained separated from the treatment allocation process and were not involved in animal feeding or sample collection, ensuring the integrity of blinding. Observation began when the bolus was regurgitated into the oral cavity and ended upon completion of swallowing, ensuring precise enumeration of chews per bolus. Monitoring was conducted twice weekly, with each animal observed for three consecutive rumination events per session. The mean number of chews per bolus during rumination was then calculated for each animal.

### 2.3. Assessment of Abnormal Feces, Horn-Base Inflammation, Hoof Redness, and Excessive Salivation

An optimized fecal consistency scoring system [27] (Table 1), adapted from established methods and tailored for local fattening cattle, was utilized in this study. Scores of ≥4 indicated dry-hard feces, while scores of ≤2 indicated loose-soft feces; both were classified as abnormal feces. Assessments were conducted by a designated veterinarian who was blinded to group assignment, using a fixed-point observation protocol. For each session, ten animals were randomly selected from each group, and fecal consistency was scored two hours after both morning and evening feedings. Observations were performed twice weekly to ensure data continuity. In addition, veterinarians conducted weekly assessments to record the incidence of clinical signs, including excessive salivation, horn-base inflammation, and hoof redness, across all experimental animals. All assessors were blinded to treatment allocation throughout the study period.

### 2.4. Collection and Processing of Samples

On day 142, prior to morning feeding, 30 mL of jugular venous blood was collected from 10 randomly selected animals in each group. A 0.2 mL aliquot was transferred to EDTA anticoagulant tubes for complete blood count analysis using a five-part differential automated hematology analyzer (BC-5800, Mindray Bio-Medical Electronics Co., Ltd., Shenzhen, China). Additionally, 1 mL of blood was placed into heparin sodium tubes, centrifuged at 3000 rpm for 3 min, and the supernatant was collected. The remaining blood was left to stand for 30 min, then centrifuged at 3000 rpm for 8 min, and the resulting serum was transferred into 1.5 mL tubes and stored at −80 °C under refrigeration until further analysis.

After slaughter, rumen fluid was collected by filtering ruminal contents through four layers of gauze, and 20 mL was aliquoted into 2 mL cryotubes. Tissue specimens (2 cm × 2 cm) from the ruminal ventral sac were collected and fixed in 4% paraformaldehyde. Rumen ventral epithelial microbiota samples were collected following the protocol described by Qingmiao Ren et al. [28]. The residual tissue was bluntly dissected to isolate the epithelium, which was then minced, divided into five 2 mL cryotubes, flash-frozen in liquid nitrogen, and stored at −80 °C. Tissue samples for subsequent omics sequencing were collected from five animals in each group, with samples from the Con group numbered AR1–5 and those from the AMC group numbered CR1–5. During transcriptome analysis, sample AR5 failed quality control; therefore, all subsequent analyses were performed using samples AR1–4 from the Con group and CR1–5 from the AMC group.

### 2.5. Enzyme-Linked Immunosorbent Assay (ELISA)

Rumen epithelium tissue was homogenized in sterile, pre-chilled saline at a ratio of 1:9 (*w*/*v*) using a tissue homogenizer to obtain a 10% (*w*/*v*) homogenate. The supernatant was collected for subsequent analyses. The concentrations of inflammatory cytokines, including interleukin-1β (IL-1β; Cat. No. H002-1-2), interleukin-6 (IL-6; Cat. No. H007-1-2), and tumor necrosis factor-α (TNF-α; Cat. No. H052-1-2), as well as oxidative stress markers, including malondialdehyde (MDA; Cat. No. A003-1), total antioxidant capacity (T-AOC; Cat. No. A015-2-1), glutathione peroxidase (GSH-Px; Cat. No. A005-1), superoxide dismutase (SOD; Cat. No. A001-3), and catalase (CAT; Cat. No. A007-7-1), were measured in both serum and rumen epithelial supernatant using commercial ELISA kits (Nanjing Jiancheng Bioengineering Institute, Nanjing, China) according to the manufacturer’s protocols. Histamine (HIS; Cat. No. H171-1-2) levels were determined in serum and rumen fluid, while lipopolysaccharide (LPS; Cat. No. E039-1-1) concentrations were measured in plasma and rumen fluid using ELISA kits from the same manufacturer. All kits have been validated by the manufacturers and are suitable for use with bovine serum, rumen fluid, and rumen epithelial homogenate samples [17,29].

### 2.6. Histopathological Analysis

Fixed ruminal tissues were rinsed, trimmed, and processed through graded ethanol dehydration, followed by xylene clearing and paraffin embedding. Sections were cut at a thickness of 5 μm with a Leica RM2235 microtome, mounted on slides, deparaffinized, and stained with hematoxylin-eosin (HE). After subsequent dehydration and clearing, coverslips were affixed with neutral resin. Entire tissue sections were systematically scanned under a light microscope, and representative photomicrographs of prominent pathological alterations were captured using a digital imaging system (Olympus BX61VS, Olympus Corporation, Tokyo, Japan).

### 2.7. Transcriptome Sequencing and Analysis

A total of 10 rumen tissues (AR1–5 from the Con group and CR1–5 from the AMC group) were randomly selected and sent to Beijing Novogene Co., Ltd. (Beijing, China) for transcriptome sequencing to characterize their gene expression profiles. Briefly, total RNA was extracted from rumen epithelial tissue samples using an RNA extraction mini kit (Qiagen, Hilden, Germany), and RNA integrity was assessed using the RNA Nano 6000 Assay Kit of the Bioanalyzer 2100 system (Agilent Technologies, Santa Clara, CA, USA). Total RNA was used for library construction. mRNA was purified with poly-T magnetic beads, fragmented at elevated temperature with divalent cations, and first-strand cDNA was synthesized from random hexamers using M-MuLV Reverse Transcriptase, followed by second-strand synthesis with DNA Polymerase I and RNase H. After end repair, adenylation, and adapter ligation, 370–420 bp fragments were selected with AMPure XP beads. Libraries were PCR-amplified with Phusion High-Fidelity DNA Polymerase and index primers, purified, and quality-checked on an Agilent Bioanalyzer 2100. Clustering of index-coded samples was performed on a cBot Cluster Generation System with TruSeq PE Cluster Kit v3-cBot-HS (Illumina) per the manufacturer’s instructions. After cluster generation, libraries were sequenced on an Illumina NovaSeq platform to yield 150 bp paired-end reads. The resulting RNA-Seq FASTQ files were aligned to the bovine genome (http://ftp.ensembl.org/pub/release-105/fasta/bos_taurus/ (accessed on 15 January 2025)) using the Hisat2 (v2.0.5) algorithm [30]. Binary alignment/map (BAM) files generated from the alignments were processed with Cufflinks to estimate transcript abundance and to detect potential isoforms. StringTie (v1.3.3b) was used to assemble mapped reads for each sample in a reference-guided approach [31]. Transcript expression was quantified as counts, and principal component analysis (PCA) was performed using these count values. Differentially expressed genes (DEGs) between groups at each time point were identified using the DESeq2 (V3.20) package in the RStudio (V4.4.3) environment. KEGG and GO enrichment analyses of the DEGs were conducted with the ClusterProfiler package in the RStudio environment. Due to the failure of sample AR5 during quality control, this sample was excluded from sequencing, resulting in final sample sizes of N = 4 for the Con group and N = 5 for the AMC group. Although this imbalance may introduce potential bias in differential expression analysis, the DESeq2 algorithm used in this study incorporates a model that is relatively robust to unequal sample sizes by estimating dispersion across genes and applying shrinkage estimators. Nevertheless, the results should still be interpreted with appropriate caution.

### 2.8. Real-Time Quantitative Reverse Transcription PCR (RT-qPCR) Analysis

Approximately 20 mg of rumen epithelium tissue was excised and placed into 1 mL of TransZol reagent (TransGen Biotech, Beijing, China). The tissue was subsequently homogenized into a uniform slurry using an automatic grinder (MB-LD48S, Beijing Boaojuhe Technology Co., Ltd., Beijing, China). After centrifugation at 4 °C and 12,000× *g* for 15 min, 1 mL of the supernatant was transferred to a new tube. Then, 200 μL of chloroform was added, and the mixture was vortexed vigorously, placed on ice for 3 min, and then centrifuged again at 4 °C and 12,000× *g* for 15 min. Subsequently, 500 μL of the supernatant was transferred to a new tube, and 500 μL of isopropanol was added. The mixture was mixed gently and incubated on ice for 10 min, followed by another centrifugation at 4 °C and 12,000× *g* for 10 min. To the resulting pellet, 700 μL of 75% ethanol was added, mixed gently, and centrifuged at 4 °C and 13,000× *g* for 10 min. The ethanol was discarded, and the mixture was centrifuged again at 4 °C and 13,000× *g* for 5 min. The residual ethanol was carefully removed with a pipette, and the sample was placed on ice in a clean hood to air dry for about 3 min. Then, 150 μL of DEPC water was added to each tube to dissolve the total RNA. RNA purity was verified by Nanodrop spectrophotometry (Thermo Fisher Scientific, Madison, WI, USA), ensuring A260/280 ratios between 1.8 and 2.0. After reverse transcription using a commercial kit (TransGen Biotech), quantitative PCR was performed with PerfectStart^®^ Green qPCR SuperMix using the following protocols: initial denaturation at 94 °C for 30 s, followed by 45 cycles of 94 °C for 5 s and 60 °C for 30 s. Relative gene expression levels were calculated using the 2^−ΔΔCq^ method, with β-actin serving as the housekeeping gene. Gene-specific primers used in this study are listed in Table 2.

### 2.9. Immunoblotting Analysis

The rumen epithelium tissue was placed in a mortar precooled with liquid nitrogen and ground into a powder. A total of 50 mg of rumen epithelium powder was dissolved in 500 μL of RIPA lysis buffer (R0010, Solarbio, Beijing, China) and 50 μL of PMSF (P1045, Beyotime Biotechnology, Shanghai, China). The mixture was sonicated, and the supernatant was collected after centrifugation. The supernatant was diluted 50-fold, and the protein concentration was measured using a BCA assay kit (P0010S, Beyotime Biotechnology, Shanghai, China). After adjusting the sample to a final concentration of 4 μg/μL, Western blot analysis was performed. The procedure was as follows: SDS-PAGE (12%), transfer to membrane (4 °C, 300 mA, 75 min), blocking with blocking solution, overnight incubation with primary antibody at 4 °C, incubation with secondary antibody at room temperature for 1 h, and imaging. The primary antibodies used were Claudin 1 Rabbit mAb (CLDN1) (#A21971, 1:1000, ABclonal, Wuhan, China), Anti-Occludin antibody (OCLN) (#Ab216327, 1:5000, Abcam, Cambridge, UK), ZO1 Rabbit Polyclonal Antibody (TJP1) (#ER41204, 1:2500, Huabio, Hangzhou, China), and β-Actin Rabbit mAb (#AC026, 1:10,000, ABclonal, Wuhan, China); the secondary antibody was HRP-conjugated Goat anti-Rabbit IgG (#AS014, 1:10,000, ABclonal, Wuhan, China). Images were captured with a full-function imaging system (Touch Imager, Shanghai eBiotrade Technology Co., Ltd., China), and band gray values were analyzed using ImageJ software (V10.8).

### 2.10. Metagenomic Sequencing and Analysis

Microbial samples were transported to Novogene Co., Ltd. (Beijing, China) within 72 h on dry ice for metagenomic sequencing, using sample identifiers consistent with those in the transcriptomics study. For species annotation, the gene catalogue was aligned with the Micro NR database to obtain species annotations for each gene, which were then integrated with the gene abundance table to generate species abundance profiles across different taxonomic levels. Principal coordinates analysis (PCoA) was conducted based on Bray–Curtis distance to assess differences in community composition, and the quality of the PCoA results was evaluated using permutational multivariate analysis of variance (PERMANOVA). Functional annotation was performed by referencing common databases, including KEGG (http://www.genome.jp/kegg/ (accessed on 16 April 2025)) for pathway annotation, eggNOG (http://eggnog5.embl.de (accessed on 16 April 2025)) for orthologous gene clusters, and CAZy (https://www.cazy.org/ (accessed on 16 April 2025)) for carbohydrate-active enzymes, enabling comprehensive functional annotation and abundance analysis. Based on species and functional abundance data, MetagenomeSeq and Linear discriminant analysis Effect Size (LEfSe) were used for multivariate statistical analyses. Comparative analysis of metabolic pathways was performed to identify differences in species and functional composition between groups.

### 2.11. Metabolomic Profiling and Analysis

Untargeted metabolomic sequencing and analysis were conducted by Biotree Biotech Co., Ltd. (Shanghai, China) to characterize the metabolite profiles in rumen fluid, using sample identifiers consistent with those in the transcriptomics study. Specifically, 50 μL of each sample was mixed with 200 μL of extraction solution (methanol:acetonitrile = 1:1, v/v, including internal standards), shaken at 750 rpm for 5 min, allowed to settle for 5 min, and then filtered through a protein precipitation plate to collect the filtrate. The raw data were converted to mzXML format using ProteoWizard and processed with an in-house R program based on XCMS for feature detection, extraction, alignment, and integration. Metabolite identification was performed using the R package and BiotreeDB (V3.0). The final dataset, containing feature number, sample name, and normalized feature area, was imported into SIMCA 18.0.1 (Sartorius Stedim Data Analytics AB, Umeå, Sweden) for multivariate analysis. Data were scaled and log-transformed to minimize noise and variance. An OPLS-DA (orthogonal projections to latent structures-discriminant analysis) approach was used to filter out orthogonal variables in metabolites that were not related to the classification variable, and non-orthogonal and orthogonal variables were analyzed separately to obtain more reliable inter-group differences in metabolites. Enrichment analysis of differentially expressed metabolites (DE Metas) was conducted using KEGG (http://www.genome.jp/kegg/ (accessed on 15 January 2025)) and MetaboAnalyst 6.0 (http://www.metaboanalyst.ca/). Correlation analysis between up- and downregulated DE Metas was conducted using an R package (V0.89); hierarchical clustering was performed with the complete linkage method, and distance was computed using Euclidean distance.

### 2.12. Statistical Analysis

All descriptive data are presented as mean ± standard deviation (SD). Data analyses were performed using SPSS 29 software (IBM; Armonk, NY, USA), Novomagic (https://magic.novogene.com (accessed on 15 January 2025)), and Lims2 (https://biotree.lims2.com (accessed on 15 January 2025)). Field data, including single rumination counts, fecal abnormality scores, horn-base inflammation, incidences of hoof redness, and salivation, were analyzed using a generalized linear mixed model (GLMM). In this model, each field parameter served as the dependent variable, with “Time” (morning or afternoon) and “Day” as fixed factors. “Day” was treated as a repeated-measures factor with a first-order autoregressive [AR(1)] covariance structure, and individual cattle were considered random effects. Factors with *p* < 0.05 were considered to have significant effects on the outcomes. Statistical analyses for white blood cell count, ELISA, RT-qPCR data and immunoblotting analysis were as follows: parametric data were analyzed using Student’s *t*-test, while nonparametric data were analyzed using the Mann–Whitney U test. GLMM, *t*-test, and Mann–Whitney U test all considered differences to be significant at *p* < 0.05. For transcriptome analysis, PCA was performed, and a PERMANOVA test was used to determine significant differences. DEGs were defined as those with *p* < 0.05 and |log2(FoldChange)| > 1, while significant enrichment for KEGG and GO pathways was defined by *p* < 0.05. In microbiome analysis, differences in relative abundance at each taxonomic level were considered significant at *p* < 0.05. For metabolomics analysis, DE Metas were identified based on variable importance in projection (VIP) > 1 and *p* < 0.05. It should be noted that the *p* values reported for differential expression and pathway enrichment analyses of transcriptomic, metabolomic, and metagenomic data are unadjusted for multiple comparisons. Unless otherwise specified, all figures were generated using GraphPad Prism 10.1.2 (GraphPad Software, San Diego, CA, USA) and Adobe Illustrator 2024 (Adobe Systems Incorporated; San Jose, CA, USA).

## 3. Results

### 3.1. Clinical Health Assessment During the Fattening Phase in Cattle

Throughout the 142-day fattening period, the AMC group exhibited a significantly greater number of individual rumination bouts than the Con group (*p* < 0.001) (Figure 1F). Continuous clinical monitoring revealed significantly lower incidences of horn-base inflammation (*p* < 0.001), hoof redness (*p* < 0.001), excessive salivation (*p* = 0.002), and abnormal feces (*p* < 0.001) in the AMC group compared to the Con group (Figure 1B–E). These clinical indicators exhibited a pattern of initial decrease followed by an increase between days 91 and 119 of the experiment. PCA and Anosim analyses indicated similar white blood cell count profiles between the two groups (*p* = 0.87) (Figure 1G). Specifically, the AMC group exhibited upward trends in lymphocyte count (LYM) and lymphocyte percentage (LYM %), which were 19.04% (5.44 ± 1.08 vs. 2.57 ± 1.00) and 16.30% (56.29 ± 11.47% vs. 48.40 ± 7.20%) (*p* = 0.08) higher, respectively, as well as a downward trend in basophil percentage (BAS%), decreasing by 36.07% (0.39 ± 0.28% vs. 0.61 ± 0.28%) (*p* = 0.10) compared to the Con group. No other hematological parameters differed between groups (*p* > 0.10) (Table 3).

### 3.2. Inflammation and Antioxidant Levels in Circulating Blood

**Figure 2:**
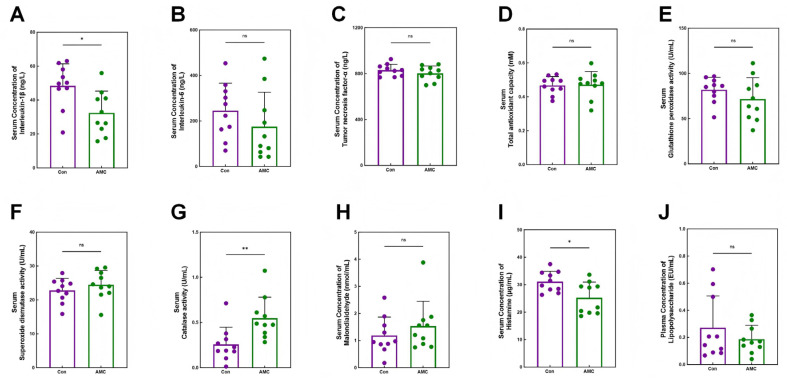
Serum index analysis and detection of blood histamine and lipopolysaccharide. (**A**–**J**): Bar plots demonstrating the serum concentrations of interleukin-1β (**A**), interleukin-6 (**B**), and tumor necrosis factor-α (**C**); (**D**–**H**): Bar plots depicting the activities of serum total antioxidant capacity (**D**), glutathione peroxidase (**E**), superoxide dismutase (**F**), catalase (**G**), and malondialdehyde (**H**); (**I**,**J**): Bar plots showing the concentrations of serum histamine (**I**) and plasma lipopolysaccharide (**J**). In (**A**–**J**), data are presented as mean ± standard deviation and were analyzed using Student’s *t*-test. Asterisks indicate significance levels (ns, *p* > 0.05; *, *p* < 0.05; **, *p* < 0.01).

The AMC group exhibited a 33.12% lower serum IL-1β concentration compared to the Con group (32.41 ± 12.91 ng/L vs. 48.46 ± 12.91 ng/L; *p* = 0.012) (Figure 2A). Although serum IL-6 and TNF-α concentrations were numerically reduced by 28.58% (175.20 ± 150.81 ng/L vs. 245.31 ± 120.76 ng/L) and 3.24% (803.61 ± 63.39 ng/L vs. 830.55 ± 51.19 ng/L), respectively, these differences were not statistically significant (*p* ≥ 0.226) (Figure 2B,C). CAT activity was markedly elevated in the AMC group, with an increase of 111.54% (0.55 ± 0.23 U/mL vs. 0.26 ± 0.19 U/mL; *p* = 0.007) (Figure 2G), while SOD activity showed a modest, non-significant increase of 7.37% (24.48 ± 4.20 U/mL vs. 22.80 ± 3.59 U/mL; *p* = 0.352) (Figure 2F). Serum HIS concentration was significantly reduced in the AMC group (25.27 ± 5.72 μg/mL vs. 31.13 ± 3.76 μg/mL; *p* = 0.015) (Figure 2I), and plasma LPS concentration was 29.63% lower (0.19 ± 0.10 EU/mL vs. 0.27 ± 0.24 EU/mL; *p* = 0.314) (Figure 2J).

### 3.3. Histopathological Alterations in Ruminal Epithelium

**Figure 3:**
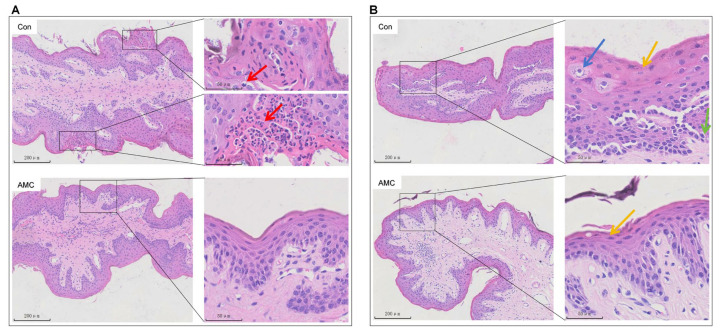
Representative hematoxylin-eosin (HE)-stained sections of ruminal epithelium. (**A**): Inflammatory cell infiltration in the rumen epithelium; (**B**): Pathological changes of rumen epithelial cells. Inflammatory cell infiltration (red arrow); granular cell swelling (blue arrows); parakeratosis of keratinized cells (yellow arrows); basal cell hyperplasia (green arrows). Left panels: 10× magnification (scale bar = 200 μm); Right panels: 40× magnification (scale bar = 50 μm).

Histological examination revealed inflammatory cell infiltration in the ruminal epithelium of the Con group (Figure 3A). Further assessment showed that the Con group exhibited more extensive parakeratosis, with increased layer thickness and broader distribution of the stratum corneum, whereas the AMC group showed less severe and limited lesions (Figure 3B). Additionally, mild swelling of granular layer cells was frequently observed in the Con group but occurred only occasionally and was less pronounced in the AMC group (Figure 3B).

### 3.4. Gene Expression Profiles of Ruminal Epithelium

**Figure 4:**
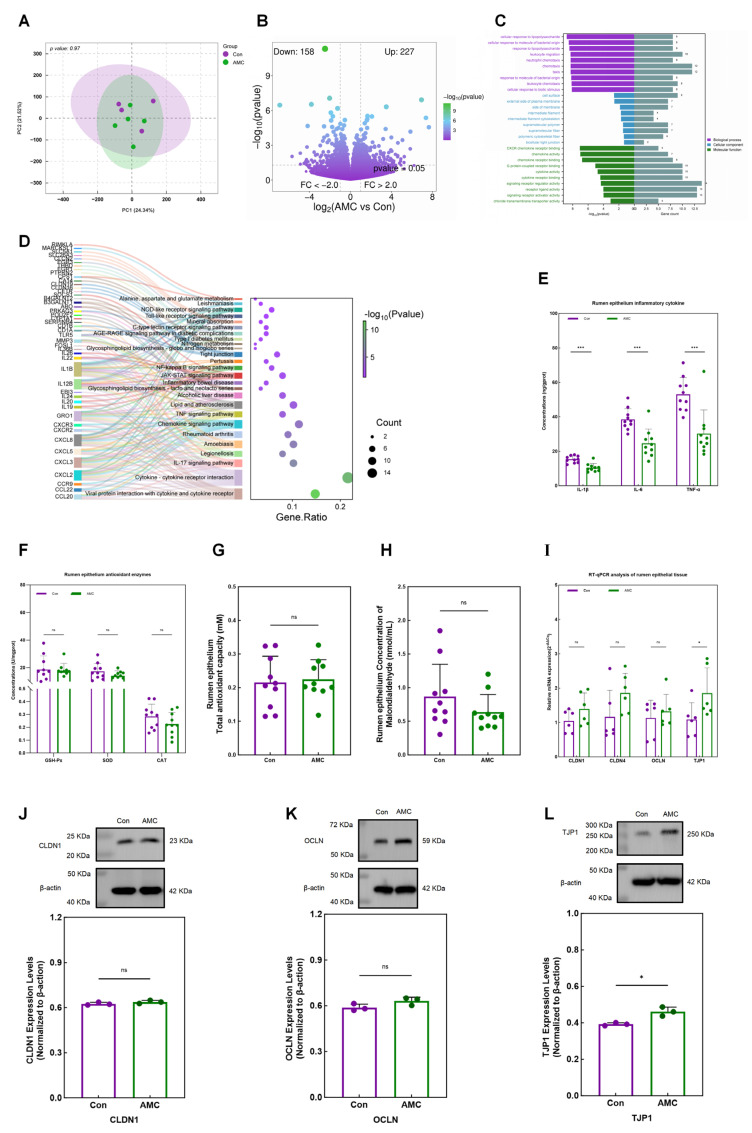
Effects of AMC on the rumen epithelium and its gene expression profiles. (**A**): Principal component analysis score plot showing the gene expression profiles in the Con and AMC groups; (**B**): Volcano plot illustrating the significantly upregulated (green) and downregulated (purple) genes between the AMC and Con groups (AMC vs. Con), with differentially expressed genes defined as those with |log_2_(FoldChange)| > 1 and *p* < 0.05; (**C**): Grouped bar plot displaying the top 10 most significantly enriched Gene Ontology terms in each category for differentially expressed genes between the AMC and Con groups, with numbers to the right of each bar indicating the number of differentially expressed genes annotated to the corresponding pathway; (**D**): Bubble Sankey diagram showing significantly enriched KEGG pathways identified by differentially expressed genes between the AMC and Con groups, with “Gene. Ratio” representing the proportion of differentially expressed genes annotated to each pathway relative to the total, and symbol colors indicating the corresponding −log10(pvalue); (**E**,**H**): Bar plot showing the supernatant concentrations of interleukin-1β, interleukin-6, tumor necrosis factor-α (**E**), as well as malondialdehyde (**H**) in each group; (**F**,**G**): Bar plot displaying the activities of glutathione peroxidase, superoxide dismutase, catalase (**F**), as well as total antioxidant capacity (**G**) in rumen epithelial tissues from each group; (**I**): Bar plot showing the relative expression levels of CLDN1, CLDN4, OCLN, and TJP1 genes in each group. (**J**–**L**): Bar plots showing the relative expression levels of CLDN1 (**J**), OCLN (**K**), and TJP1 (**L**) proteins in each group. In (**E**–**L**), data are presented as mean ± standard deviation and were analyzed using Student’s *t*-test (**E**–**H**,**J**–**L**) or Mann–Whitney test (**I**). Asterisks indicate significance levels (ns, *p* > 0.05; *, *p* < 0.05; ***, *p* < 0.001).

RNA-seq generated 438,966,876 raw reads and 428,862,656 clean reads, with an average of 7.32 ± 0.73 G raw bases and 7.15 ± 0.72 G clean bases per sample (Supplementary Table S2). After assembly, a total of 21,883 genes were annotated and quantified across nine samples (Supplementary Table S3). PCA and PERMANOVA revealed similar gene expression profiles between the groups (*p* = 0.97; Figure 4A). DESeq2 identified 385 DEGs between the AMC and Con groups, with 227 upregulated and 158 downregulated in the AMC group (Figure 4B and Supplementary Table S4). GO enrichment analysis of the DEGs revealed significant enrichment in 180 GO terms, including 121 biological processes (BP; *p* < 0.05), nine cellular components (CC; *p* ≤ 0.047), and 50 molecular functions (MF; *p* ≤ 0.047) (Supplementary Table S5), with the top 10 GO terms presented in Figure 4C. KEGG enrichment analysis identified 26 significantly enriched pathways (Log2(FoldChange) > 1 and *p* ≤ 0.045), including viral protein interaction with cytokine and cytokine receptor, cytokine–cytokine receptor interaction, IL-17 signaling pathway, Legionellosis, amoebiasis, rheumatoid arthritis, chemokine signaling pathway, TNF signaling pathway, and lipid and atherosclerosis (Figure 4D and Supplementary Table S6).

Comparative analysis of gene expression related to tissue inflammation, antioxidant indices, and tight junctions within enriched KEGG pathways by RNA-seq showed that the concentrations of IL-1β, IL-6, and TNF-α in the rumen epithelial tissue were significantly lower in the AMC group (*p* < 0.001) (Figure 4E). ELISA revealed similar activities of GSH-Px, SOD, CAT, and T-AOC in the rumen epithelium tissue between the Con and AMC groups (Figure 4F,G). Additionally, the MDA concentration in the AMC group decreased by 26.44% (0.64 ± 0.26 mmol/mL vs. 0.87 ± 0.48 mmol/mL), but this decrease was not statistically significant (*p* = 0.202) (Figure 4H). RT-qPCR analysis further demonstrated a significant increase in TJP1 gene expression in the AMC group (*p* = 0.026), while the expression levels of CLDN1 (*p* = 0.225), CLDN4 (*p* = 0.093), and OCLN (*p* = 0.937) genes were similar between the Con and AMC groups (Figure 4I). This result was also validated by immunoblotting analysis (Figure 4J–L), showing a significantly higher TJP1 protein expression in the AMC group than in the Con group (*p* = 0.010), while the protein expression of CLDN1 (*p* = 0.212) and OCLN (*p* = 0.096) did not differ significantly.

### 3.5. Profiling the Composition and Structure of the Rumen Epithelial Mucosal Microbiome

**Figure 5:**
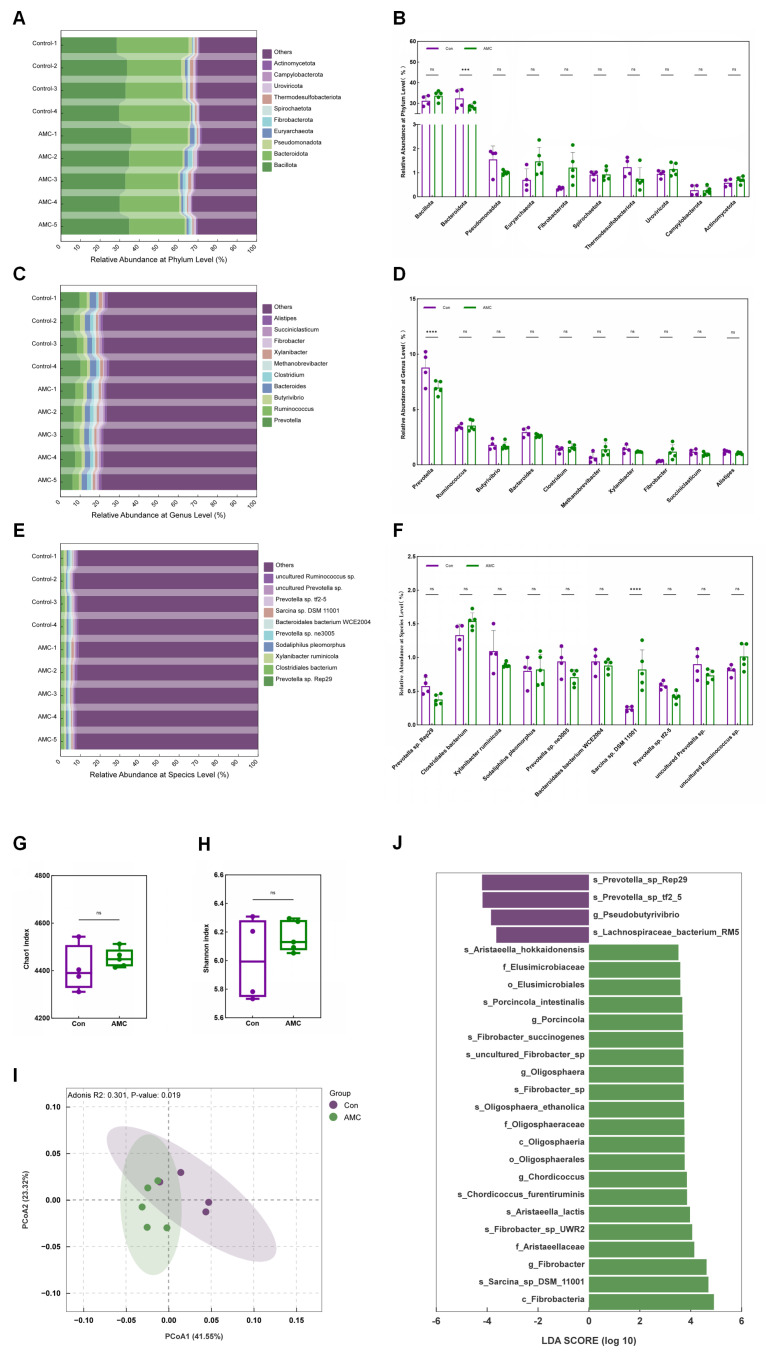
Effects of AMC on the composition and structure of the ruminal epithelial mucosal microbiota. (**A**,**C**,**E**): Stacked bar plots showing the relative abundance of taxa at the phylum (**A**), genus (**C**), and (E) species levels for each sample; (**B**,**D**,**F**): Bar plots depicting the relative abundance of the top 10 abundant taxa at the phylum (**B**), genus (**D**), and species (**F**) levels for each group; (**G**,**H**): Bar plots showing the Shannon and Chao1 indices of the ruminal epithelial mucosal microbiota for each group; (**I**): Principal coordinates analysis score plot based on Bray–Curtis distance, showing the ruminal epithelial mucosal microbiota in each group; (**J**): Bar plot showing the identified bacterial biomarkers in each group using Linear Discriminant Analysis (LDA) Effect Size, with thresholds of *p* < 0.05 and |LDA| > 3.5. In (**B**,**D**,**F**,**G**,**H**), data are presented as mean ± standard deviation and were analyzed using the Wilcoxon rank-sum test (**B**,**D**,**F**) or Student’s *t*-test (**G**,**H**). Asterisks indicate significance levels (ns, *p* > 0.05; ***, *p* < 0.001); ****, *p* < 0.0001.

The average raw base total yield of rumen epithelial mucosal microbial samples was 13.07 ± 2.16 G. After quality filtering, the average clean data volume was 12.91 ± 2.13 G, with 98.51 ± 0.14% of bases having a quality value above Q20 (Supplementary Table S7). As shown in Figure 5A,B, the dominant microbial phyla in the rumen epithelium were *Bacteroidota* (Con group: 32.39 ± 4.63%, AMC group: 28.36 ± 1.39%) and *Bacillota* (Con group: 31.27 ± 2.51%, AMC group: 33.75 ± 2.35%), with *Bacteroidota* abundance significantly lower in the AMC group compared to the Con group (*p* = 0.0013). At the genus level, *Prevotella* (Con: 8.80 ± 1.50%, AMC: 6.98 ± 0.60%) and *Ruminococcus* (Con: 3.41 ± 0.24%, AMC: 3.55 ± 0.48%) were predominant, and *Prevotella* abundance was highly significantly lower in the AMC group compared to the Con group (*p* < 0.0001) (Figure 5C,D). At the species level, the dominant species in the Con group were *Clostridiales bacterium* (1.33 ± 0.17%), *Xylanibacter ruminicola* (1.09 ± 0.31%), and *Prevotella* sp. *ne3005* (0.94 ±0.21%), while in the AMC group, *Clostridiales bacterium* (1.54 ± 0.12%), *uncultured Ruminococcus* sp. (1.01 ± 0.19%), and *Xylanibacter ruminicola* (0.89 ± 0.04%) were the most abundant. Notably, the abundance of *Sarcina* sp. *DSM 11001* was significantly higher in the AMC group compared to the Con group (*p* < 0.0001) (Figure 5E,F).

As shown in Figure 5G,H, there were no significant differences in alpha diversity between the Con group and the AMC group (*p* > 0.05). However, PCoA based on Bray–Curtis distance at the genus level revealed significant differences in the composition of the ruminal epithelial mucosal microbiota between groups (R2 = 0.301, *p* = 0.019; Figure 5I). LEfSe analysis identified 25 bacterial taxa with significantly different abundance: 21 were enriched in the AMC group and 4 in the Con group (Figure 5J). The main marker genera for the Con group included *Pseudobutyrivibrio*, *Prevotella_sp_Rep29*, *Lachnospiraceae_bacterium_RM5*, and *Prevotella_sp_tf2_5*, while the AMC group was primarily characterized by *Fibrobacter* (LDA > 3.5, *p* < 0.05).

### 3.6. Functional Characterization of the Ruminal Epithelial Mucosal Microbiome

**Figure 6:**
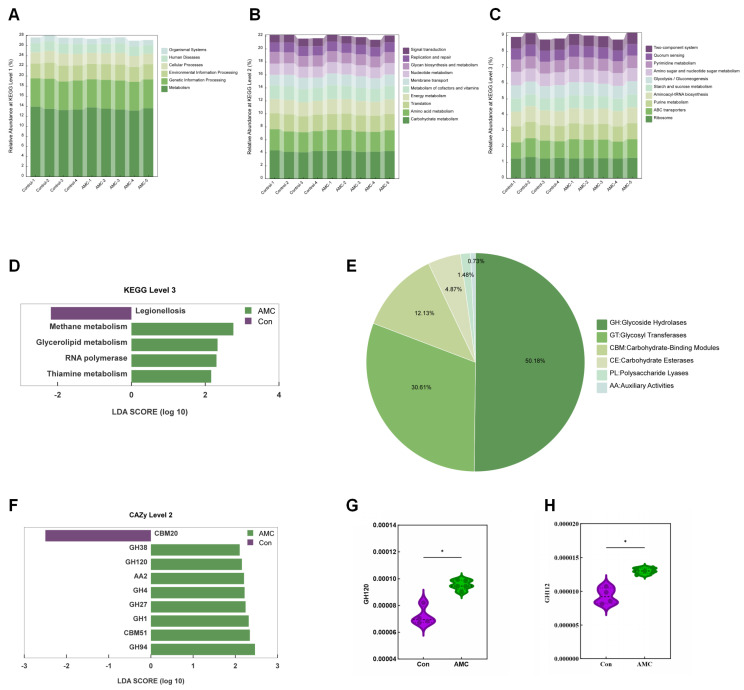
Effects of AMC on the function of the ruminal epithelial mucosal microbiota. (**A**–**C**): Bar plots showing the relative abundances of KEGG functional annotations at levels 1, 2, and 3, respectively, for each sample; (**D**): Bar plot displaying the identified KEGG functional biomarkers at level 3 in each group using Linear Discriminant Analysis (LDA) Effect Size, with thresholds of *p* < 0.05 and |LDA| > 2.0; (**E**): Pie chart illustrating the proportion of each functional category of the ruminal epithelial mucosal microbiota at CAZy level 1; (**F**): Bar plot showing the identified functional biomarkers at level 2 in each group using Linear Discriminant Analysis (LDA) Effect Size, with thresholds of *p* < 0.05 and |LDA| > 2.0; (**G**,**H**): Violin plots presenting the abundances of CAZyme level 2 functions, GH112 (**H**) and GH120 (**G**), with statistical analysis performed using Mann–Whitney U test. Asterisks indicate significance levels (ns, *p* > 0.05; *, *p* < 0.05).

According to KEGG database annotation, at the first level, most genes were assigned to Metabolism, followed by Genetic Information Processing, Environmental Information Processing, Cellular Processes, Human Diseases, and Organismal Systems (Figure 6A). At the second level, the top 10 annotations by relative abundance included Carbohydrate metabolism, Amino acid metabolism, Translation, Energy metabolism, Metabolism of cofactors and vitamins, Nucleotide metabolism, Membrane transport, Glycan biosynthesis and metabolism, Replication and repair, and Signal transduction (Figure 6B). At the third level, the top 10 annotations were ko03010 (Ribosome), ko02010 (ABC transporters), ko00230 (Purine metabolism), ko00970 (Aminoacyl-tRNA biosynthesis), ko00520 (Amino sugar and nucleotide sugar metabolism), ko00010 (Glycolysis/Gluconeogenesis), ko00500 (Starch and sucrose metabolism), ko00240 (Pyrimidine metabolism), ko02020 (Two-component system), and ko02024 (Quorum sensing) (Figure 6C). LEfSe analysis of the level 3 KEGG functional annotations revealed significant enrichment of pathways such as ko00680 (Methane metabolism), ko00561 (Glycerolipid metabolism), ko03020 (RNA polymerase), and ko00730 (Thiamine metabolism) in the AMC group, while ko05134 (bacterial, Legionellosis) was significantly enriched in the Con group (LDA > 2.0, *p* < 0.05) (Figure 6D).

For CAZyme analysis, the protein sequences of non-redundant genes were annotated into six categories of carbohydrate-active enzymes (Figure 6E). Glycoside Hydrolases (GHs) constituted the largest proportion at 50.18%, followed by Glycosyl Transferases (GTs) at 30.61%, Carbohydrate-Binding Modules (CBMs) at 12.13%, and Carbohydrate Esterases (CEs) at 4.87%. Polysaccharide Lyases (PLs) and Auxiliary Activities (AAs) represented 1.48% and 0.73%, respectively. LEfSe analysis revealed that the AMC group was significantly enriched in CAZy level 2 functional annotations, including GH94, CBM51, GH1, GH27, GH4, AA2, GH120, and GH38, whereas CBM20 was predominantly enriched in the Con group (LDA > 2, *p* < 0.05; Figure 6F). Furthermore, MetaStat analysis demonstrated that the AMC group exhibited significantly higher abundances of CAZyme level 2 genes annotated to GH112 (*p* = 0.016) and GH120 (*p* = 0.016) compared to the Con group (Figure 6G,H).

### 3.7. Ruminal Fluid Metabolome Profiling

**Figure 7:**
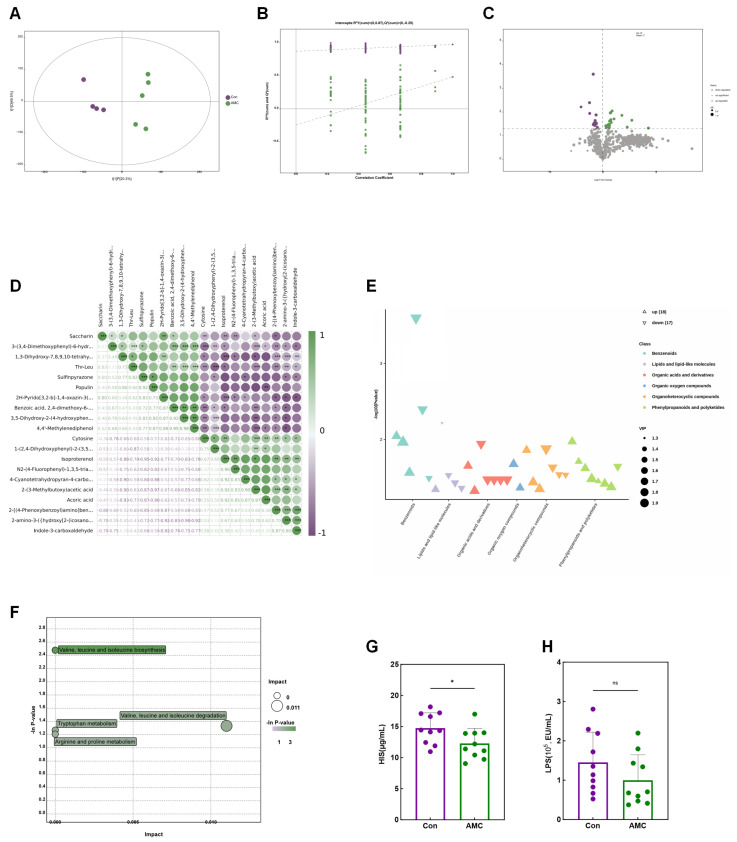
Effects of AMC on the ruminal fluid metabolome. (**A**): Orthogonal partial least squares discriminant analysis score plot showing the metabolic profiles of ruminal fluid from the Con and AMC groups; (**B**): A permutation test scatter plot was created, and the model’s robustness was validated with 200 permutation tests; (**C**): Volcano plot illustrating the metabolites with significantly different abundances between the Con and AMC groups; (**D**): Bubble heatmap presenting the correlations between the top 10 upregulated and downregulated differentially expressed metabolites between the AMC group and the Con group (AMC vs. Con); (**E**): Bubble chart illustrating the classification categories of the differentially expressed metabolites; (**F**): Bubble chart showing the four key KEGG pathways significantly enriched by the differentially expressed metabolites; (**G**,**H**): Bar plots demonstrating the concentrations of histamine (HIS) (**G**) and lipopolysaccharide (LPS) (**H**) in ruminal fluid from each group, with data expressed as mean ± standard deviation and statistical significance analyzed using Student’s *t*-test. Asterisks indicate significance levels (ns, *p* > 0.05; *, *p* < 0.05; **, *p* < 0.01; ***, *p* < 0.001).

A total of 1084 metabolites were identified in the non-targeted liquid chromatography–mass spectrometry (LC-MS) metabolomics analysis (Supplementary Table S8). Significant differences in ruminal fluid metabolites between the Con and AMC groups of fattening beef cattle were observed, as indicated by the OPLS-DA model and permutation test scatter plots (Figure 7A,B), with all samples falling within the 95% Hotelling T2 ellipse. The overall distribution of differential metabolites was illustrated using a volcano plot (Figure 7C), from which metabolites with VIP > 1 and *p* < 0.05 were selected for intergroup analysis. Compared to the Con group, the AMC group exhibited 18 upregulated and 17 downregulated metabolites (Supplementary Table S9). Correlation analysis of the top 10 upregulated and downregulated metabolites revealed a negative correlation between upregulated and downregulated metabolites (Figure 7D). These DE Metas were classified into six categories, including Benzenoids (6), Lipids and lipid-like molecules (5), Organic acids and derivatives (7), Organic oxygen compounds (2), Organoheterocyclic compounds (7), and Phenylpropanoids and polyketides (8) (Figure 7E).

To elucidate the mechanisms underlying altered metabolic pathways, enrichment analysis was conducted on the DE Metas. Four key pathways were identified as most significantly associated with the observed metabolic changes through enrichment and topological analyses, including Valine, leucine and isoleucine degradation; Valine, leucine and isoleucine biosynthesis; Tryptophan metabolism; and Arginine and proline metabolism (Figure 7F). Subsequently, metabolomics indicators related to KEGG pathway enrichment were compared in rumen samples. The results showed that, compared with the Con group, the AMC group had a significantly reduced HIS content in rumen fluid (*p* = 0.038) and a 31.01% decrease in LPS content (100,171.33 ± 64,475.96 EU/mL vs. 145,187.03 ± 77,116.63 EU/mL; *p* = 0.174) (Figure 7G,H).

### 3.8. Integrated Analysis of the Ruminal Epithelial Mucosal Microbiome, Ruminal Fluid Metabolome, and Ruminal Epithelial Transcriptome

**Figure 8:**
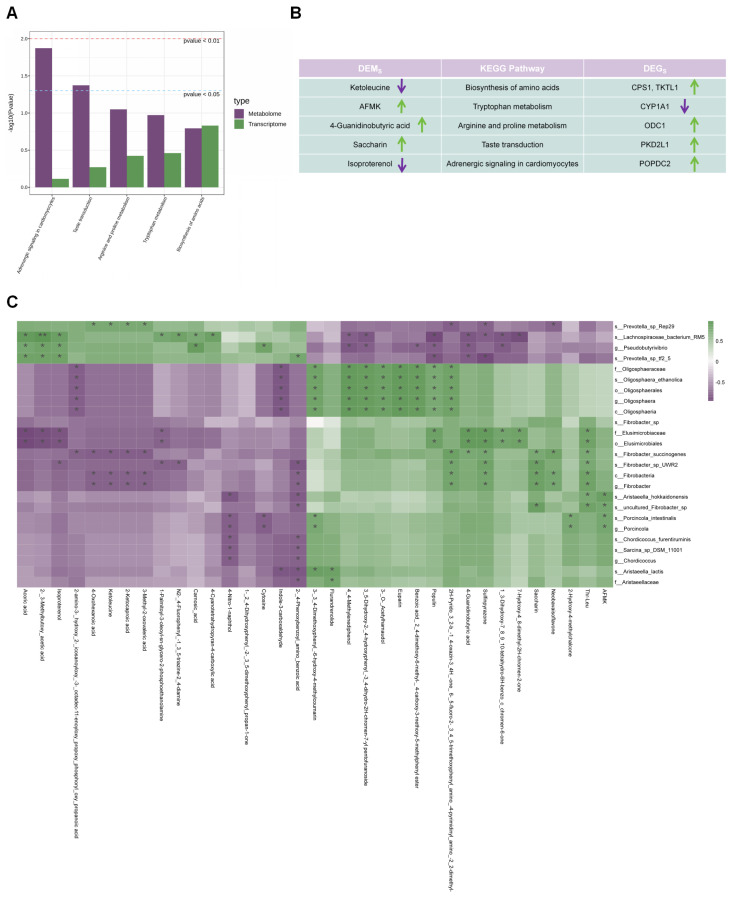
Interrelationships among the rumen epithelial mucosal microbiome, ruminal fluid metabolome, and rumen epithelial transcriptome. (**A**): Grouped bar plot showing the shared KEGG pathways co-enriched by differentially expressed genes in rumen epithelium and differentially expressed metabolites in ruminal fluid. Bar colors indicate data categories (green for differentially expressed genes, purple for differentially expressed metabolites); higher *Y*-axis values (−log10(*p*-value)) represent greater statistical significance; (**B**): Table providing detailed information about the differentially expressed metabolites and genes within the co-enriched KEGG pathways identified from the rumen epithelial transcriptome and ruminal fluid metabolome; green arrows indicate upregulation, while purple arrows indicate downregulation; (**C**): Heatmap illustrating the Spearman correlations between signature microbiota in the rumen epithelial mucosa and ruminal metabolites. Green squares represent positive correlations, while purple squares represent negative correlations. Asterisks “*” indicate significant correlations (|r| > 0.7, *p* < 0.05). The bar plot in (**A**) was generated using Metware Cloud (https://cloud.metware.cn) (accessed on 29 September 2025), a free online platform for data analysis. The heatmap in (**C**) was generated using the Wekemo Bioincloud (https://www.bioincloud.tech) (accessed on 29 September 2025). Asterisks indicate significance levels (*, *p* < 0.05; **, *p* < 0.01).

Integrated analysis of DEGs and DE Metas identified co-enrichment in several KEGG pathways, including Biosynthesis of amino acids, Tryptophan metabolism, Arginine and proline metabolism, Taste transduction, and Adrenergic signaling in cardiomyocytes (Figure 8A) (Supplementary Tables S10 and S11). Figure 8B illustrates the upregulation and downregulation patterns of DE Metas and DEGs within these commonly enriched pathways. Spearman correlation analysis between the ruminal epithelial mucosal microbial markers (LDA > 3.5) and differential metabolites in the rumen fluid revealed strong associations, particularly within the AMC group. Notably, fiber-degrading bacteria such as *Fibrobacter* and *Porcincola* exhibited significant correlations with key differential metabolites (*p* < 0.05, |r| ≥ 0.7) (Figure 8C). Downregulated metabolites, including ketoleucine in the co-enriched pathways of the epithelial transcriptome and metabolome, showed significant negative correlations with marker bacteria such as *Fibrobacter_succinogenes*, *Fibrobacteria*, and *Fibrobacter* (*p* < 0.05, |r| ≥ 0.7). Isoproterenol also demonstrated a significant negative correlation with *Fibrobacter_sp_UWR2* (*p* < 0.05, |r| ≥ 0.7). Conversely, upregulated metabolites such as AFMK were significantly positively correlated with these marker bacteria, including *uncultured_Fibrobacter_sp*, *Aristaeella_hokkaidonensis*, *Porcincola_intestinalis*, and *Porcincola* (*p* < 0.05, |r| ≥ 0.7). Additionally, 4-guanidinobutyric acid showed significant positive correlations with multiple fiber-degrading bacteria, such as *Fibrobacter_succinogenes* (*p* < 0.05, |r| ≥ 0.7). Saccharin was also positively correlated with these bacteria and other Fibrobacter species, including *uncultured_Fibrobacter_sp*, *Fibrobacter_succinogenes*, *Fibrobacter_sp_UWR2*, *Fibrobacteria*, and *Fibrobacter* (*p* < 0.05, |r| ≥ 0.7).

**Figure 9:**
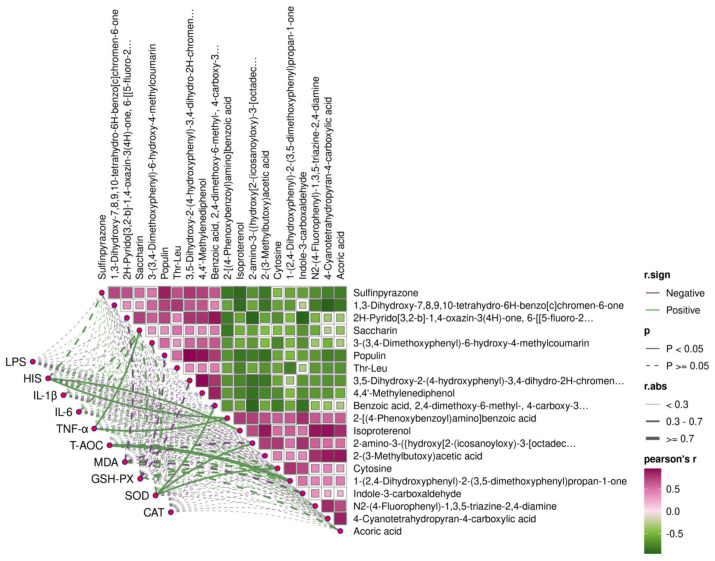
Spearman correlation analysis of metabolites and phenotypes. Green solid lines represent positive correlations, while purple solid lines represent negative correlations. The advanced correlation analysis was performed using the OmicStudio tools at https://www.omicstudio.cn/tool (accessed on 29 September 2025).

As shown in Figure 9, Spearman correlation analysis between the top 10 DE Metas in rumen fluid and phenotypic indicators revealed several significant associations. Specifically, in rumen fluid, HIS was positively correlated with the downregulated metabolites 2-[(4-Phenoxybenzoyl)amino]benzoic acid and isoproterenol (*p* ≤ 0.041, 0.3 < |r| < 0.7). In rumen epithelial tissue, TNF-α showed significant positive correlations with the upregulated metabolite saccharin and the downregulated 2-[(4-Phenoxybenzoyl)amino]benzoic acid (*p* ≤ 0.037, 0.3 < |r| <0.7). T-AOC was significantly positively correlated with the downregulated metabolite 1-(2,4-Dihydroxyphenyl)-2-(3,5-dimethoxyphenyl)propan-1-one (*p* = 0.004, |r| ≥ 0.7). SOD exhibited significant positive correlations with the upregulated benzoic acid, 2,4-dimethoxy-6-methyl-, 4-carboxy-3-methoxy-5-methylphenyl ester, as well as the downregulated metabolites 2-amino-3-({hydroxy [2-(icosanoyloxy)-3-[octadec-11-enoyloxy]propoxy}phosphoryl}oxy)propanoic acid and cytosine (*p* ≤ 0.047, 0.3 < |r| < 0.7).

## 4. Discussion

Although high-concentrate diets can improve short-term growth performance in ruminants, the subsequent accumulation of volatile fatty acids leads to a decrease in ruminal pH, directly compromising the structural and functional integrity of the rumen epithelial barrier [32,33]. An intact rumen epithelium is essential for efficient absorption and robust barrier function, which are fundamental for maintaining rumen health and productivity.

Throughout the 142-day feedlot period, clinical indicators were systematically evaluated using a standardized scoring system administered exclusively by a trained veterinarian according to established protocols, ensuring consistency and minimizing subjective variation. Results demonstrated that cattle supplemented with AMC exhibited significantly higher individual rumination bouts, along with significantly lower incidences of horn-base inflammation, hoof redness, excessive salivation, and abnormal feces, as well as trends toward improved lymphocyte and basophil percentages, reflecting better clinical health status in the AMC group compared to the Con group (Figure 1B–F, Table 3). These clinical indicators declined from day 91 to day 119 and then rose. This pattern occurred because the concentrate-to-forage ratio was adjusted on day 91 based on herd health, but the original diet structure was restored on day 105 while the AMC dose remained unchanged. Consequently, Figure 1B–E reflects this trend; nevertheless, the AMC group’s health remained consistently better than that of the Con group. Previous research has demonstrated that saliva generated through chewing possesses buffering capacity, which is critical for rumen health [34,35]. Thus, monitoring mastication activity serves as a practical approach for early detection of ruminal acidosis. These findings indicate that AMC supplementation can enhance rumen homeostasis and overall health by increasing chewing activity, thereby stimulating saliva secretion and elevating rumen pH. However, it should be acknowledged that production performance data, including average daily gain, dry matter intake, and feed efficiency, were not collected in the present study. Therefore, further investigation is needed to determine whether the observed improvements in chewing activity and clinical health translate into enhanced digestion and growth performance in finishing beef cattle. Fecal consistency was evaluated as a key indicator of nutritional management and digestive efficiency [36], and the incidence of abnormal fecal conditions in AMC group cattle was significantly reduced, further indicating improved rumen health. This was confirmed by the reduction in inflammatory cytokines in the body (Figure 2A–C) and the histological examination of the epithelium (Figure 3A,B).

Transcriptional features related to inflammation, epithelial development, and metabolism showed associations with rumen epithelial characteristics. In this study, high-throughput RNA-Seq analysis was used to identify DEGs and perform KEGG functional enrichment in the rumen epithelial tissue of fed cattle. DEGs between the AMC and Con groups were significantly enriched in pathways such as Tight junction, Chemokine signaling pathway, Nitrogen metabolism, and Viral protein interaction with cytokine and cytokine receptor (Figure 4D), all of which are associated with epithelial barrier function and immune responses. Notably, the AMC group exhibited upregulation of several chemokine- and cytokine-related genes, including *CCL20*, *CCL22*, *CXCL2*, *CXCL3*, *CXCL5*, *IL19*, *IL20*, and *IL24* (Figure 4D). Although some chemokine-related genes are typically associated with inflammatory responses, histological examination revealed that the rumen epithelium of AMC-supplemented cattle remained structurally intact with minimal inflammatory infiltration, suggesting that the observed gene expression changes may instead reflect enhanced immune surveillance or homeostatic regulation. For example, upregulation of the *CCL* family genes affects immune system function; *CCL22* is constitutively expressed under homeostatic conditions and can be induced during inflammation to enhance immune response efficiency and bolster host defense against pathogens [37]. Thus, the upregulation of such genes in the AMC group may indicate a role in immune modulation. However, it is important to acknowledge that the sample size for the omics analyses in this study was relatively small, which may limit statistical power and increase the risk of false-positive findings. Therefore, although these findings provide preliminary insights, further studies with larger cohorts and prospective power analysis are warranted to establish causality and validate the results presented here. *IL19*, *IL20*, and *IL24*, members of the IL-10 cytokine family, possess anti-inflammatory and immunoregulatory functions [38]. Specifically, *IL19* can reduce adhesion molecule expression and leukocyte infiltration [39], while *IL20* participates in anti-inflammatory responses and mucosal healing, promoting epithelial innate immune responses and thus limiting damage from viral and bacterial infections [40]. Additionally, enrichment of the epithelial tight junction pathway, with upregulation of *CD1A* and *CD1B* genes (Figure 4D), underscores the importance of these genes in rumen epithelial immune function. Differential levels of inflammatory cytokines, antioxidant enzyme activities, and tight junction proteins between the AMC and Con groups were validated by ELISA, RT-qPCR and immunoblotting analyses (Figure 4E–L), supporting the transcriptomic findings. Collectively, these results suggest that AMC supplementation is associated with enhanced rumen epithelial immune responses, maintenance of barrier integrity, and improved rumen health.

Our previous study demonstrated that alkaline mineral water (AMW) administration modulates the nasopharyngeal microbiota in transported calves [17]. Building on this, we investigated the impact of AMC supplementation on the ruminal epithelial mucosal microbiota of fattening beef cattle using rumen metagenomics. Specifically, *g_Fibrobacter* sp. was identified as one of the biomarker species. The ruminal epithelial mucosal microbiota protects against harmful microorganisms by competing with pathogens and forming defensive biofilms, and its composition is sensitive to dietary changes and host regulation, directly influencing barrier function and local immune responses [41,42]. KEGG pathway analysis indicated that the epithelial mucosa microbiota in the Con group was significantly enriched in pathogenic pathways, whereas the AMC group showed marked enrichment in beneficial metabolic pathways related to energy production, lipid metabolism, fundamental cellular functions, and vitamin synthesis (Figure 6D). CAZy functional annotation further demonstrated that AMC supplementation was associated with enhanced carbohydrate degradation capabilities in the ruminal epithelial mucosal microbiota, as reflected by the enrichment of genes encoding fibrolytic and amylolytic enzymes (e.g., GH94 and GH1) and substrate-binding modules (e.g., CBM51, AA2). These changes suggest a potential shift in the functional profile of the microbial community—from a greater reliance on easily degradable starch (as inferred from the enrichment of CBM20 in the Con group) toward an increased capacity for the decomposition of complex plant polysaccharides. Such functional remodeling may not only improve host nutrient acquisition but also strengthen the epithelial biological barrier through GT-mediated biofilm synthesis, thereby potentially optimizing rumen health through both metabolic and protective mechanisms. Interestingly, these microbial findings align with the observed activation of epithelial tight junction pathways and increased expression of TJP1 protein in the AMC group. This convergence of microbiota and host transcriptomic data provides a mechanistic perspective on how AMC-induced shifts in microbial structure may be linked to enhanced epithelial barrier function and improved rumen health.

Rumen metabolomics provides insights into the interaction mechanisms between the rumen host, microbes, and their metabolites. Major rumen metabolites, particularly organic acids, are mainly produced by rumen microbes digesting the basal diet [43]. Coupled with metagenomic analyses, these findings suggest that AMC supplementation may modulate the rumen metabolite profile by altering the mucosal-associated microbiota on the rumen epithelium, as further supported by changes in rumen fluid HIS and LPS concentrations (Figure 7G–H). Notably, multiple studies have shown that tryptophan metabolism can regulate the gut microbiota and alleviate mucosal damage [44,45]. Based on these findings, we hypothesize that AMC may exert potential effects on tryptophan metabolism in ruminants.

Subsequently, we integrated multi-omics data with phenotypic indicators and found that, in the AMC group, associated genes were predominantly enriched in Metabolic pathways, particularly in tryptophan metabolism and arginine and proline metabolism (Figure 8A). Previous research has shown that proline can alleviate intestinal inflammation in mice and help maintain gut homeostasis [46]. Metabolites derived from tryptophan, such as 5-hydroxy-L-tryptophan, indole-3-propionic acid, and indole-3-acetic acid, are effective in restoring intestinal structural integrity and permeability, thereby supporting epithelial barrier function and reducing inflammation [47]. Further correlation analyses revealed that upregulated DE Metas, such as sulfinpyrazone, Thr-Leu, and 4-guanidinobutyric acid, possess anti-inflammatory effects [48,49,50] and are positively correlated with *Fibrobacter* in the AMC group (Figure 8C). This finding suggests that *Fibrobacter* fermentation may be associated with the production of anti-inflammatory metabolites and the suppression of ruminal epithelial inflammation. AMC supplementation was also associated with upregulation of metabolites such as AFMK, 4-guanidinobutyric acid, saccharin, and Thr-Leu, all of which showed positive correlations with fiber-degrading bacteria. Notably, AFMK is a potent antioxidant that may accumulate in response to activated tryptophan metabolism mediated by fiber bacteria [51], while the enrichment of 4-guanidinobutyric acid (an arginine metabolite) and saccharin suggests that the microbiota may influence host nitrogen cycling through nitrogen metabolism pathways. Positive correlations between rumen fluid HIS and epithelial TNF-α with DE Metas like 2-[(4-Phenoxybenzoyl)amino]benzoic acid and isoproterenol (Figure 9) suggest that these metabolites may participate in mucosal immune responses by reducing HIS release or modulating pro-inflammatory pathways. *Fibrobacter*, identified as a biomarker genus in the AMC group, promotes colonization of the epithelial mucosa and shows a positive association with sulfinpyrazone and a negative association with *Prevotella*. Given that *Fibrobacter* is involved in cellulose degradation, while *Prevotella*, a Gram-negative genus, may produce harmful metabolites such as LPS when overgrown [52,53], shifts in their relative abundance may have differential effects on rumen health. However, several limitations of this study should be acknowledged. The sample size for the omics analyses (transcriptomics, metagenomics, and metabolomics) was relatively small (N = 4–5 per group), which may limit statistical power and increase the risk of false-positive or false-negative findings. Moreover, the *p* values reported for differential expression and pathway enrichment analyses were unadjusted for multiple comparisons, further compounding this issue. Therefore, these findings should be considered preliminary and exploratory, and future studies with larger sample sizes and the application of more rigorous statistical methods (e.g., FDR) are warranted to validate the results presented here. In summary, the present study provides evidence that AMC supplementation is associated with improved clinical health outcomes and rumen epithelial integrity in fattening beef cattle. The observed changes in ruminal microbiota, metabolite profiles, and host gene expression suggest a coordinated response that may contribute to enhanced rumen health. However, given the correlational nature of these findings, the proposed mechanistic pathways, including the involvement of tryptophan metabolism, anti-inflammatory metabolites, and microbial shifts, should be considered hypotheses requiring direct validation in future intervention studies.

## 5. Conclusions

**Figure 10:**
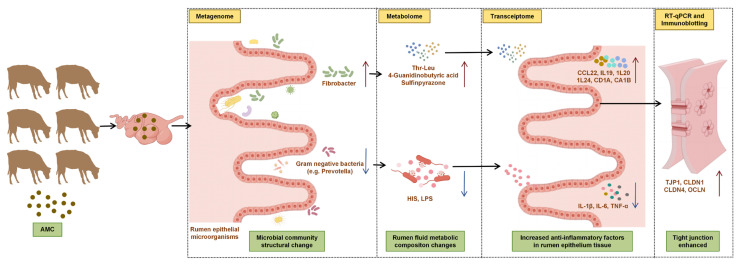
Mechanisms underlying the effects of AMC on fattening cattle. AMC supplementation alters the composition of the ruminal epithelial mucosal microbiota, characterized by an increased abundance of fiber-degrading bacteria and a simultaneous decrease in Gram-negative bacteria, such as *Prevotella*. This microbial shift leads to significant changes in the rumen fluid metabolite profile: anti-inflammatory metabolites such as sulfinpyrazone, Thr-Leu, and 4-guanidinobutyric acid display positive correlations with fiber-degrading bacteria and negative correlations with Prevotella. The decrease in Gram-negative bacteria results in lower concentrations of lipopolysaccharide (LPS) and histamine (HIS) in the rumen fluid, collectively reducing inflammatory stimulation of rumen epithelial cells. Upregulation of IL-10 family genes, including IL-19, IL-20, and IL-24, enhances the anti-inflammatory capacity of epithelial cells. Additionally, upregulation of genes related to tight junctions increases the integrity of the rumen epithelial barrier function. Red arrows indicate increases; blue arrows indicate decreases.

This study demonstrated that dietary AMC supplementation during the fattening phase was associated with improved overall health and rumen function in beef cattle. The observed benefits appeared to be mediated through coordinated changes in the ruminal microbiota, metabolite profiles, and host epithelial gene expression. Specifically, AMC supplementation increased the abundance of fibrolytic bacteria such as *Fibrobacter*, which may enhance fiber degradation efficiency, while reducing the relative abundance of Gram-negative genera including *Prevotella*, potentially lowering endotoxin load within the rumen. These microbial shifts were accompanied by elevated levels of anti-inflammatory metabolites, including sulfinpyrazone, Thr-Leu, and 4-guanidinobutyric acid, and reduced concentrations of HIS and LPS, suggesting a potential role for these changes in mitigating inflammatory stimulation of the epithelial barrier. At the host level, upregulation of IL-10 family cytokines (*IL19*, *IL20*, *IL24*) and activation of tight junction pathways were also correlated with enhanced epithelial integrity and immune homeostasis (Figure 10). In conclusion, this study identifies associations between AMC supplementation and multiple indicators of improved rumen health in beef cattle. The findings point to potential mechanistic links involving ruminal microbe–metabolite–host interactions that collectively contribute to the observed health outcomes. However, given the correlational nature of this study, the proposed mechanisms should be considered hypotheses requiring direct validation in future intervention studies. These insights may inform the development of targeted nutritional strategies to support sustainable practices in the beef industry.

## Figures and Tables

**Table 1:** Detailed criteria for fecal consistency scoring.

Score	Morphological Characteristics
1	porridge-like, watery, mucoid, and foul-smelling
2	loose and shapeless, less than 2.5 cm thick, with bubbles present
3	mound-shaped, with a central depression and 2–4 concentric rings
4	mound-shaped, 5–12 cm thick, without a central depression, containing undigested feed particles
5	hard, pellet-shaped, dark-colored, and odorless

**Table 2:** Detailed information on the primers used in the RT-qPCR assay.

Gene	Primer (5′-3′)	Accession Number
β-actin	F: CCATCGGCAATGAGCGGTTC R: AGCACCGTGTTGGCGTAGAG	NM_173979.3
CLDN1	F: CGTGCCTTGATGGTGAT R: CTGTGCCTCGTCGTCTT	NM_001001854.2
CLDN4	F: TCATCGGCAACATCGTCAC R: CAGCAGCGAGTCGTACACCTTG	NM_001014391.2
OCLN	F: GAACGAGAAGCGACTGTATC R: CACTGCTGCTGTAATGAGG	NM_001082433.2
TJP1	F: TTGGACAAAGAGAAGGGTGAGA R: AGACCAACCGTCAGGAGTCA	XM_024982006.2

**Table 3:** White blood cell parameters.

Items	Con	AMC	*p* Value
WBC (10^9^/L)	9.44 ± 1.62	9.76 ± 1.29	0.63
NEU (10^9^/L)	3.84 ± 0.93	3.27 ± 1.68	0.37
NEU% (%)	40.57 ± 6.92	32.77 ± 14.90	0.15
LYM (10^9^/L)	4.57 ± 1.00	5.44 ± 1.08	0.08
LYM% (%)	48.40 ± 7.20	56.29 ± 11.47	0.08
MON (10^9^/L)	0.78 ± 0.17	0.89 ± 0.39	0.43
MON% (%)	8.33 ± 1.26	9.37 ± 4.73	0.51
EOS (10^9^/L)	0.20 ± 0.16	0.12 ± 0.08	0.17
EOS% (%)	2.09 ± 1.68	1.18 ± 0.78	0.14
BAS (10^9^/L)	0.06 ± 0.03	0.04 ± 0.03	0.19
BAS% (%)	0.61 ± 0.28	0.39 ± 0.28	0.10
NLR	0.88 ± 0.31	0.64 ± 0.34	0.13

WBC, white blood cell count; NEU, neutrophil count; NEU%, NEU ratio; LYM, lymphocyte count; LYM%, LYM ratio; MON, monocyte count; MON%, MON ratio; EOS, eosinophil count; EOS%, EOS ratio; BAS, basophil count; BAS%, BAS ratio; NLR, NEU-to-LYM ratio. Data are expressed as mean ± standard deviation and statistically analyzed using *t*-tests. *p* value ≤ 0.1 was considered to indicate a trend toward significance, and *p* < 0.05 was considered statistically significant.

## Data Availability

The raw transcriptome sequence data have been uploaded to the NCBI SRA database (BioProject: PRJNA1346806 at https://dataview.ncbi.nlm.nih.gov/object/PRJNA1346806?reviewer=lnkeahlqf2mbq507n951o6cbre (accessed on 20 October 2025)); the raw mucosal microbiome metagenomic sequence data have been uploaded to the NCBI SRA database (BioProject: PRJNA1346885 at https://dataview.ncbi.nlm.nih.gov/object/PRJNA1346885?reviewer=7arfhilrrpr3mmhidtesc9phgb (accessed on 20 October 2025)); the raw rumen fluid non-targeted metabolomics sequence data have been uploaded to the MetaboLights database (MTBLS13166; https://www.ebi.ac.uk/metabolights/reviewerf956a54f-631f-469b-9000-99515ea88604 (accessed on 18 October 2025)).

## Supplementary Materials

The following supporting information can be downloaded at: https://www.mdpi.com/article/10.3390/ani16060992/s1, Table S1: Composition and nutrient levels of total mixed rations; Table S2: Statistical overview of the raw data generated from the transcriptome sequencing of rumen epithelium; Table S3: Summary of annotated and quantified genes across nine samples; Table S4: Summary of differentially expressed genes (DEGs) between the AMC and Con groups; Table S5: Summary of significantly enriched GO terms of differentially expressed genes (DEGs). Table S6: Summary of 26 significantly enriched KEGG pathways; Table S7: Statistical overview of the original data of the rumen epithelial mucosal microbiota obtained through metagenomic sequencing; Table S8: List of identified metabolites in non-targeted LC-MS metabolomics analysis; Table S9: List of 35 differentially expressed metabolites (18 upregulated and 17 downregulated) in the AMC group compared to the Con group; Table S10: Summary of KEGG pathways co-enriched with DEGs identified from metabolomics data; Table S11: Summary of KEGG pathways co-enriched with DE Metas identified from transcriptomics data. Table S12: D91–D104 Beef Cattle Basal Diet Composition and Nutritional level; Table S13: The components and ion concentrations in AMC.

## Abbreviations

The following abbreviations are used in this manuscript:

AMC	Alkaline Mineral Complex
LPS	Lipopolysaccharide
HIS	Histamine
DEGs	Differentially Expressed Genes
DE Metas	Differentially Expressed Metabolites
RT-qPCR	Real-Time Quantitative Reverse Transcription PCR
