# Supplementary material for: Multi-Omics Analysis Reveals That Alkaline Mineral Complex Reshapes Rumen Mucosal Microbiota and Metabolites and Enhances Rumen Epithelial Barrier Function in Fattening Cattle
Source: Animals (Basel). 2026 Mar 22;16(6):992. doi: 10.3390/ani16060992 (PMC13024318; doi:10.3390/ani16060992)
Supplement: Supplementary file 1 [file animals-16-00992-s001.zip › Supplementary Table S1.pdf]

Supplementary Table S1: Composition and Nutrient Levels of Total Mixed Rations

Diet Composition	Content (%)
DDGS	40.88
Corn	26.80
Soybean meal	1.81
Premix ①	1.79
Wheat bran	1.83
Rapeseed dregs	1.86
$NaHCO_3$	0.80
NaCl	0.40
Whole corn silage	14.47
Hay	9.36
Total	100.00

Nutrient levels	Content (%)
NE_{mf}/(MJ/Kg) ②	1.44
CP	13.79
NDF	40.04
peNDF	19.65
TDN	64
Ca	0.29
P	0.33

① Each kilogram of premix contains the following: 6,000 - 300,000 IU of Vitamin A, 6,000 - 90,000 IU of Vitamin D3, 200 mg of Vitamin E, 1,000 - 5,000 mg of Zinc, 1,000 - 6,000 mg of Iron, 275 - 750 mg of Copper, and 600 - 4,000 mg of Manganese. ② NEmf and TDN were calculated based on the Beef Cattle Feeding Standard (NY/T 815-2004), the others are measured values. ③ Crude protein (CP) was determined using the Kjeldahl method. The NDF and peNDF contents were determined based on the methods described in the national standard for determination of neutral detergent fiber in feeds (GB/T 20806-2022) and the local standard for determination of physically effective neutral detergent fiber in feeds (DB61/T 1063-2017), respectively. Calcium and phosphorus were analyzed using inductively coupled plasma optical emission spectrometry (ICP-OES) after wet ashing.
