# Supplementary material for: Multi-Omics Analysis Reveals That Alkaline Mineral Complex Reshapes Rumen Mucosal Microbiota and Metabolites and Enhances Rumen Epithelial Barrier Function in Fattening Cattle
Source: Animals (Basel). 2026 Mar 22;16(6):992. doi: 10.3390/ani16060992 (PMC13024318; doi:10.3390/ani16060992)
Supplement: Supplementary file 1 [file animals-16-00992-s001.zip › Supplementary Table S13.pdf]

Supplementary Table S13: The components and ion concentrations in AMC

Microbial strain	Calculate Concentration (mg/kg)
Bacillus subtilis	5×10^7 CFU / g
Bacillus licheniformis	1×10^8 CFU / g

Ions	Calculate Concentration (mg/kg)
Na^+	27482.00
K^+	25103.00
Zn^+	5.20
$Ge4^+$	0.13

All values are calculated concentrations.
