# Supplementary material for: Multi-Omics Analysis Reveals That Alkaline Mineral Complex Reshapes Rumen Mucosal Microbiota and Metabolites and Enhances Rumen Epithelial Barrier Function in Fattening Cattle
Source: Animals (Basel). 2026 Mar 22;16(6):992. doi: 10.3390/ani16060992 (PMC13024318; doi:10.3390/ani16060992)
Supplement: Supplementary file 1 [file animals-16-00992-s001.zip › Supplementary Table S2.pdf]

Supplementary Table S2: Statistical overview of the raw data generated from the transcriptome sequencing of rumen epithelium

Sample Name	Raw Reads (#)	Raw Bases (G bp)	Clean Reads (#)	Clean Bases (G bp)	Error Rate	Q20 (%)	Q30 (%)	GC Content (%)
Contorl-1	45868392	6.88G	44538648	6.68G	0.01	98.96	96.77	52.35
Control-2	52585934	7.89G	51532594	7.73G	0.01	99	96.91	51.04
Control-3	55907082	8.39G	54765442	8.21G	0.01	98.98	96.86	51.05
Control-4	42806520	6.42G	41801670	6.27G	0.01	98.96	96.79	51.25
AMC-1	47042218	7.06G	45805368	6.87G	0.01	99.06	97.06	52.34
AMC-2	48331038	7.25G	47340444	7.1G	0.01	99.09	97.18	51.11
AMC-3	54098022	8.11G	52827616	7.92G	0.01	99.02	96.96	51.65
AMC-4	42085708	6.31G	41155978	6.17G	0.01	98.99	96.87	52.16
AMC-5	50241962	7.54G	49094896	7.36G	0.01	99.01	96.93	51.87
