# Supplementary material for: Multi-Omics Analysis Reveals That Alkaline Mineral Complex Reshapes Rumen Mucosal Microbiota and Metabolites and Enhances Rumen Epithelial Barrier Function in Fattening Cattle
Source: Animals (Basel). 2026 Mar 22;16(6):992. doi: 10.3390/ani16060992 (PMC13024318; doi:10.3390/ani16060992)
Supplement: Supplementary file 1 [file animals-16-00992-s001.zip › Supplementary Table S7.pdf]

Supplementary Table S7: Statistical overview of the original data of the rumen epithelial mucosal microbiota obtained through metagenomic sequencing

Sample Name	Raw Base(G)	Clean Base(G)	Clean Q20(%)	Clean Q30(%)	Clean GC(%)	Effective (%)	NonHost Base(G)
Contorl-1	12.08	11.9	98.38	95.04	50.09	98.44	11.11
Control-2	13.75	13.54	98.61	95.78	47.79	98.48	10.12
Control-3	13.78	13.55	98.55	95.61	48.44	98.32	11.92
Control-4	12.15	12	98.53	95.51	49.14	98.73	11.68
AMC-1	18.03	17.84	98.73	96.37	50.12	98.99	17.12
AMC-2	11.28	11.19	98.28	95.25	49.46	99.19	11.01
AMC-3	13.81	13.64	98.63	96.08	48.51	98.77	11.72
AMC-4	10.87	10.76	98.48	95.73	49.17	98.99	10.18
AMC-5	11.9	11.78	98.43	95.59	50.08	98.99	11.37
